# The Hospital Nursing Supplement

**Published:** 1893-06-10

**Authors:** 


### "The Hospital" Nursing Mirror.

Being the Extra Nursing Supplement of "The Hospital" Newspaper.

[Contributions for this Supplement should be addressed to the Editor, The Hospital, 428, Strand, London, W.C., and should have the word "Nursing" plainly written in left-hand top corner of the envelope.]

### News from the Nursing World.

### THE ROYAL WEDDING.

Our friends, the Pension Fund Nurses, must talk over last week's suggestion of a river picnic or sea trip, and let us know their wishes. The sooner they do so the better, and then the discussion of the necessary arrangements can be entered upon. We shall, therefore, be glad if each one of the members of the R.N.P.F. will express her personal wish with as little delay as possible.

### THE DUCHESS OF CONNAUGHT.

The prizes and certificates granted by the Portsmouth centre of the St. John Ambulance Association were distributed to the successful candidates by H.R.H. the Duchess of Connaught on the Queen's birthday. The display of skill took place on Governor's Green, and the arrangements made for the large concourse of spectators were excellent. Great interest and enthusiasm were manifested in the whole of the proceedings. The Duke of Connaught presented a challenge trophy, which will have to be won three years in succession by the same candidates before it becomes their own property.

### THE NURSE DOLLS.

With the view of making The Hospital Collection as complete as possible, and also to avoid the reproduction of the same uniforms, we shall be glad if our readers will continue to keep us informed of their proposed contributions of dolls. It is a pity to risk repetition when it can be avoided by sending a post-card to the office to inquire whether special uniforms have already been promised to the collection. We have set our minds on having a completely representative party of Nurse Dolls, which will always be available for reference. Many nurses will comprehend the practical value of an advantage in which they will freely share, and we are well aware of the cordial sympathy and support which have been already evinced by every reader interested in this particular subject. We beg to thank our numerous correspondents for their good wishes and hearty co-operation. We hope that all contributors will remember that dolls should be received at 428, Strand, by June 17th; they should be addressed to the Editor and marked "Dolls" in left hand bottom corner of address card.

### REGISTERED NURSES.

The unprofessional public seems much puzzled by the recent discussion on "trained nurses," and is led to wonder what it all means. "What a capital plan it will be for nurses to get diplomas," said a patient the other day. "To get what?" gasped the astonished nurse. "Why, certificates; that we may know whether a woman is trained or not." The nurse promptly explained to the kindly but ignorant speaker that she had possessed for years a certificate of training, and also of character, and that all regular training schools have been accustomed to grant these since their first establishment, every worker whose record is good having taken care to keep her name on the register of her own training school. Most important of all is the fact that no appointment is obtained by a qualified nurse without reference being made to that said school, whatever supplementary register she may have joined. All nurses would do well to reflect on these facts. So little is known outside hospitals of the thorough system which prevails within them, that only a working nurse, who has attained to an independent position by means of her hospital "diploma," "certificate," or "testimonial," can fully appreciate how completely the value of this outweighs any later additions she may add to the original proof of her being a trained and duly-qualified attendant for the sick.

### THE LATE DR. HADDEN.

Dr. Hadden, whose death we regretfully recorded last week, was laid to rest in West Brompton Cemetery on May 30th. There was a large gathering of doctors and many other friends, including some of the nurses belonging to the Co-operation to which he has been so invaluable a friend. A lovely wreath, representing the badge of the Co-operation, namely, a red cross on a white circle, was the offering of the nurses by whom his death is universally lamented.

### WITHOUT IMPEDIMENTS.

The enthusiasm of would-be probationers is often known to cool down, when they read the printed list of moral qualities which hospital authorities desire to find in each aspirant. Yet that long list, supplemented by a still more extensive one, of "things to be learnt," fades into insignificance beside the virtues and accomplishments expected of private nurses. One patient demands a young woman, "pleasant to look at, good-tempered and cheerful, but staid and skilful." The next requires a tall nurse "without a fringe," quiet, but not dull; age between twenty-nine and thirty. Yet another prefers fringes, but dislikes tall, fair women. A middle-aged, active woman, not too stout, but yet not thin; a religious abstainer, or an abstainer from religious conversation; all these are stipulated for, in turn, by those who employ nurses. A lady the other day specially bargained to have some one who did not wear any false teeth! Rather a delicate point to insist on in these degenerate days. It is strange to find that a question as to the appearance of a nurse generally precedes the query as to her skill. Happily this is not always the case, and opinions as to good looks vary so considerably that neither the fair nor the dark, the tall nor the dumpy woman, is exclusively favoured.

### INSTRUCTION FOR MENTAL NURSES.

Systematic instruction has been given at Warneford Asylum during the last six months by Dr. Neil, who has held separate classes for the attendants and nurses. The first course embraced general nursing, the treatment of common accidents, and elementary physiology. The second part dealt with psychology, and the special nursing of patients with mental disorders. Demonstrations and examinations were included in the course and four of the pupils subsequently obtained certificates of proficiency from the Medico-Psychological Society.

APPRECIATED INSTRUCTION.

Valuable instruction in ambulance work has been given at Morpeth. Classes have been held in the Mechanics' Institute by Drs. McDowall and Bramwell, and they have been very well attended. Amongst those who have benefited by the excellent instruction given, were many police constables and also several attendants from the County Asylum. The examinations were conducted by Dr. Anderson, of Seaton Delaval.

A HOME AT BEXHILL.

Last autumn a small Convalescent Home for Children was opened by Lady Brassey at Bexhill. She furnished and fitted up a house to accommodate eight little people. Children who are too weak and sickly for ordinary Homes will be admitted, and even small wounds do not render candidates ineligible. In the first place, patients discharged recently from hospitals will be chosen, but as long as there is a vacant bed any ailing child is considered a suitable occupant.

CORNWALL.

The Newlyn Nursing Society has issued its fourth annual report, which records excellent work done by Nurse Goddard, whose services seem thoroughly appreciated in the district. The relief fund, always a valuable adjunct to nursing, has provided necessary nourishment for the sick, and it appears to be very well managed. It was decided at the meeting that boxes should be placed in the Fisherman's Rest, the salesmen's offices, and other suitable places, to receive contributions towards the funds of the Nursing Society. The Vicar of the parish suggested that a box might also be placed in the church, distinguished by a special inscription, for the same purpose. The universal interest and sympathy exhibited in connection with the association offer good promise that the new departure will receive adequate support.

HOW TO SECURE CONVALESCENCE.

Sadberge Hall, which has been taken as a Convalescent Home, for six months, by the Stockton and Thornaby Nursing Association, will accommodate eight patients at a time. It is believed that a hundred persons will be benefited in the course of the summer. It is impossible to over-estimate the value of such a supplement to the work of the district nurses, and it is to be hoped that the committee will presently see their way to securing a permanent Convalescent Home. At the annual meeting, Mr. R. Lorraine, a working man, said, "It was a great comfort to his class to know, when they left their home in the morning, that their sick ones would be visited by the nurses of that association, who literally carried sunshine with them. With regard to the need for a Convalescent Home, he had known of people sending to Darlington for tickets to get their friends into the Convalescent Home at the seaside, which showed the need for a Convalescent Home in connection with Stockton." If this is the general feeling, there is but little fear of any failure in the carrying out of work so well begun.

EDUCATION IN DEALING WITH EMERGENCIES.

Instruction in ambulance and nursing, for the wives and daughters of miners, is likely to receive the attention which the subject deserves. In those centres where the experiment has been tried the results have been excellent, and there is no doubt that the knowledge thus acquired will enormously benefit those whose work places them hourly in positions of peculiar peril. Every woman in the land should be trained to give "first aid to the injured," but as the teaching needed for this is naturally limited, the opportunities for acquiring this minimum of skill should be made the most of. Such learning is attended by one serious drawback, namely, the inordinate amount of self-reliance which a little knowledge begets. A short, and necessarily superficial course, is not sufficient to show learners their ignorance. Details picked up at such classes may be showy, but they are, naturally, elementary. Only time can betray the smallness of the knowledge, and meanwhile grave mischief may be done. Everyone in a hospital knows that it is the newest probationer who is the boldest nurse. She would, if not restrained, during her first month, commit disastrous blunders, the bare recollection of which would cover her with dismay even six months later. Therefore it should be borne in mind that humility must be inculcated on those whose so-called training is limited to attendance on a few lectures, and whose certificate is gained after a perfunctory examination, perhaps held merely by the teacher himself. Let all amateurs understand that intelligence in dealing with emergencies ought to be mastered by every woman, but that out of such knowledge alone can never be evolved a "Trained" nurse.

SHORT ITEMS.

Nurse Pilkington has finished her first year's work amongst the sick poor at Kew, and has become a general favourite with her patients.—At the fourth annual meeting of the Wolverhampton Nursing Institute it was decided to consider the immediate erection of suitable premises, those at present in use proving insufficient. The work done by the nurses among the sick poor is reported excellent.—Two nurses have disappeared from the Statutory Hospital at Bath without leave. The Chairman of the Committee, in dealing with the matter, said, "in case the nurses applied for a reference, this would have to be taken into consideration." He might fittingly have added that a full explanation would be demanded.—The Berwick Guardians have granted £15 to the Ladies' Nursing Association, which is about to engage a second nurse.—The Chippenham Board of Guardians, having received no replies to their advertisement for a hospital nurse, have agreed to offer a higher salary, namely, £35 per annum.—It has been decided to add six bed-rooms, a bath-room, and box-room to the Trained Nurses' Institute at Bedford, also to make the needful alterations to dining-room and kitchen.—It is proposed to set up a Nurses' Home at Halifax at the beginning of August, under the same management as the one at Huddersfield, which has nearly completed its third year.—A nursing demonstration was recently given at Coggeshall by Miss Elizabeth Hill, to members of the mothers' meeting and their friends, 130 in number; the lecture was much enjoyed.

THE DISABLED NURSE.

We have received another grateful letter from the Disabled Nurse, in which she says, "May I ask you to thank all the nurses and friends who have so kindly contributed towards the allowance sent to me weekly. I cannot be grateful enough to them. The money is such a great help, and I do not know what I should have done without it, for I have been ill now over a year. The doctor seems to doubt whether I shall ever be strong enough to take up nursing again. During the last few weeks I have certainly been worse, I am sorry to say."

### Sanitation for Nurses.

By a Sanitary Inspector.

### IX.—SOME RECENT SANITARY PROVISIONS.

With the view of preventing, as far as possible, the spread of infectious diseases, various carefully defined duties have been imposed on the sanitary authority by the Public Health Act of 1891. Thus every sanitary authority is obliged, by section 59 of this Act, to provide within or without their district, proper premises, with all necessary apparatus and attendance for the proper disinfection or destruction, also for the carriage, or the provision of suitable vessels for the removal of all articles, whether bedding, clothing, &c., which have become infected by any dangerous infectious disease." Here it may be as well to call to mind that for the purposes of this Act "a dangerous infectious disease" is any of those which are scheduled or declared to be notifiable.

In England such diseases are small-pox, cholera, diphtheria, menbranous croup, erysipelas, scarlatina, typhoid fever, typhus, pueperal fever, relapsing or continued fever. Also the sanitary authority is permitted at discretion, though not compelled, to provide the same facilities for removal of clothing and disinfection in the case of any other disease. In every instance all such articles are to be removed, disinfected, and returned to the owner, or, when necessary, destroyed free of charge. If it is necessary to destroy any articles, the owner is to be compensated for their loss, and, if in the process of disinfection any "unnecessary" damage, that is damage that could have been avoided with reasonable care, has been done, then the owner shall be compensated. In the case of unavoidable damage no claim for compensation can be made. Hence, under some circumstances considerable damage may be done to articles, but no redress can be obtained if the sanitary authority can prove that due care and precaution were taken in disinfecting. On the other hand, however, and to counterbalance this apparent hardship, it must be remembered that the owner of the infected articles is spared all responsibility, thought, and expense connected with disinfection when once he has put the matter into the hands of the sanitary authority.

If infectious disease occur in a private house, an inspector will be sent on application to carry out the necessary disinfection. He will bring with him all that is required, including strips of paper and paste to fasten up doors and windows, as well as a sufficient quantity of sulphur, and the necessary apparatus for burning it. The inspector will also give full directions as to the best means of disinfecting articles which will wash, and can therefore be dipped into a liquid disinfectant of proper strength. When the owner of the house cannot afford the cost it is done free of charge.

Disinfection has very properly been placed under the supervision of the sanitary authority, as private individuals cannot be trusted to do it thoroughly and intelligently. Nevertheless, there still exists a serious source of danger, from the fact that many people are either not yet fully aware of the extreme thoroughness necessary, if disinfection is to be other than a mere farce, or else self-interest transcends in importance every other consideration. Anyhow, it is not an uncommon occurrence for articles to be surreptitiously removed from the infected room because it is feared that the process of disinfection may injure them. This is quite unpardonable, and a district nurse should bear in mind the possibility of it happening, and guard against it accordingly, either by watchfulness or by trying to imbue the owner with a due sense of responsibility. The Act imposes a fine of £10 on "any person refusing to deliver up for disinfection articles which have been exposed to infectious disease."

To meet the case of those who live in one room only, the sanitary authority is by section 60 required to provide, "free of charge, temporary shelter or house accommodation with any necessary attendants for the members of any family in which any dangerous infectious disease has appeared, who have been compelled to leave their dwellings for the purpose of making such dwellings to be disinfected by the sanitary authority."

By section 61 it is not permitted to wilfully throw, or allow anyone else to throw into an ashpit or any vessel for the reception of refuse or ashes, such as a dustbin, ash-tub, &c., any rubbish which has been infected by a dangerous infectious disease without having previously thoroughly disinfected the same. Under this head would, of course, be included such things as poultices, rags, and other applications which have been used by the patient. The owner of the house or any responsible person shall apply to the sanitary authority, who will arrange for the proper removal and disinfection or destruction of all such "rubbish" without further trouble to the owner. Neglect of this provision incurs a fine not exceeding £5, and an additional fine not exceeding £2 for every day that the offence is continued after notice has been given.

A penalty of £20 is imposed on any person who "knowingly lets for hire any house or part of a house, whether a private house or an inn, in which any person has been suffering from a dangerous infectious disease," without having it and all infected articles in it properly disinfected to the satisfaction of a properly qualified medical practitioner, or who lets, or even shows for the purpose of letting, any house or part of a house in which there has been a dangerous infectious disease within six weeks, or who makes false answers to any questions as to there having been a case of dangerous infectious disease within that time is liable to a fine of £20, or to imprisonment for a month, either with or without hard labour, at the discretion of the magistrate. Further, a fine of £10 is ordered to be imposed on the owner or any other responsible person who vacates a house in which there has been a case of dangerous infectious disease within six weeks of its occurrence, without having had the house previously thoroughly disinfected to the satisfaction of a duly qualified medical practitioner. It is now compulsory to transfer to a proper hospital any person suffering from any dangerous infectious disease who is without proper lodging or acommodation, that is, in a common lodging house, or even in a room with other persons, or who is lodged in a tent or van, or is on board a vessel. To effect such removal a certificate, duly signed by a legally qualified medical practitioner, is required, and also the consent of the superintending body of the hospital to which the patient is to be removed; but it is almost superfluous to say that both these are easily obtained. Under these circumstances the removal is to be at the expense of the sanitary authority. This most necessary and useful provision has been made compulsory by the last Public Health Act we are glad to say. In the previous Act of 1875 it was only an optional provision, though strongly recommended to the authorities. In the event of objection on the part of the patient or his friends, or if he prove himself troublesome or refractory, it is only necessary to prove to the satisfaction of a magistrate that the removal of such a person is needful for the due protection and safety of others, and an order is granted to compel his removal to a hospital.

Section 67 makes illegal the wilful exposure in any street, public place, shop, or inn of a person suffering from a dangerous infectious disease, whether such exposure be of the infected person himself, or of those in charge of him. Neither shall such an infected person "give, lend, sell, transmit, remove, or expose without having it previously disinfected to the satisfaction of a duly qualified person, any bedding, clothing, or other articles." Any infringement of this order brings a penalty of £5. Moreover, any person suffering from dangerous infectious disease who shall milk any animal, or pick fruit, or follow any occupation connected with food, or carry on any trade whatsoever in such a manner as to be likely to spread the infectious disease, shall be liable to a fine not exceeding £10.

### Hospital Nursing in the North of India.

(By Our Indian Correspondent.)

### II.—IN THE WARDS.

In the Followers' and Cantonment Hospitals the old clothing and equipment of the Station Hospitals are used up. They are given in to the Commissariat from the Station Hospital when condemned there. What is serviceable is given to these native hospitals, and the sheets, old blankets, and very ragged clothing are used by the troops for cleaning their rifles, &c. There is theoretically no waste in Government Stores, or extravagance.

Each bed in the hospital had a mattress on, and a cast-off military counterpane for an under sheet, a small pillow, and a country blanket for a covering, and Government, I believe, sanctions towels for every one of these hospitals, but natives don't use them.

The first patient I saw was a lithotomy case doing well, the lateral operation having been performed. The calculus had weighed over an ounce; the boy was about thirteen. The beds, according to native fashion, were very low; there was a wondrous array of cradles. I saw a Salter's cradle and a MacIntyre splint.

There were four or five fractures—one compound fracture of the ankle, several bad wounds, and joints briefly described by my conductor as "strumous." There was a poor old man almost a mass of abscesses. Some of the patients had, I thought, a very famine-stricken appearance, and they looked at me with their eyes, and held out their hands as if they would have begged openly if they had dared.

I believe in these hospitals, they have to a great extent to supply their own diets. Milk and rice are issued in some, and extras are sometimes allowed by the medical officer's orders.

I noticed one or two native women in the ward giving drink and food to their lords and masters.

The medical ward my conductor apparently took no interest in, though very proud of his surgical ward. I saw a case of lichen, a young native, very stout and puffy, lips thick and protruding; at first I thought he was an African. A curious effect was produced by his skin, where the spots only were black, and the rest a red bronze.

The operation theatre was light and airy, the table serviceable, the mackintoshes beautifully fresh and clean, a nice instrument cupboard, tidy and well arranged, with glass doors.

In this respect they are better off than at the Station Hospital, where the operations are performed either in the wards or the verandahs, no operation-rooms being provided.

The women's ward was in another block. There was also an eye ward, and there were some eye cases, but six women had been discharged that morning, and none were left. My conductor informed me that the services of a lady doctor from the bazaar were called in for the Purdah women, but that the Surgeon-Major attended to the rest.

This hospital struck me this same as all others into which I have been in India—it lacked nurses. No nurse visible, but patients assisting each other. In India the hospitals have their medical attendants, but where are the nurses in proportion to patients and disease? Yet I must admit that notwithstanding the lack of nursing the results are good. There may be such a thing as over nursing, but we have not got it out here. The aspect of the hospital throughout was clean and tidy, but there was a sadly hopeless expression in addition to the look of hunger on the faces, which grieved me.

I heard rather a nice story the other day, a refreshing one in this hard, practical, work-a-day world.

Two years ago, in one of the hottest native cities in India a lady doctor was living. She was working in connection with the Zenana Mission. Far away from home and kindred she had one faithful companion, the sharer of her bed and board.

They were never apart, wherever the little dog was seen it was the forerunner of his mistress.

In May of '91, a frightful epidemic of cholera broke out in the close, narrow, dirty lanes of the city. So bad was it that year that the dark yellow plague cloud left its shadow also over the luxurious houses of the Civil Lines, and swept through the clean and airy cantonments, leaving its mark behind by the empty places in the ranks of the troops, and silence in the married soldiers' quarters. Strong men, women, and children swept away.

The troops moved out into camp, but it followed them there, and there was scarcely time to dig the common pit to place the dead in. Two years are forgotten in India as soon as twenty at home, but yet there are many who tell yet how the General and the Garrison Chaplain, men widely different in religious thought, yet united together in humanity and charity, worked from morning till night amidst that stricken garrison, and how by their personal courage and example they helped to stem the panic and put a stop to the spreading of the disease.

There are but few who know of two heroes in the heart of the city, namely, the lady doctor, working mid sordid surroundings, accompanied by her faithful doggie, till she at fast fell ill of the fell disease. There her little friend lay beside her, taking no food, and in his own kind dumb way trying to comfort her, but she died. They found her with her little friend lying on her breast. He refused to let them touch her, and defended her body till he was beaten and forced off and secured while they carried her away to bury her.

Two days afterwards some one looked into her room, and there, where she had died, lay her faithful little friend, cold and still, his work also ended.

### More Work for Competent Women.

Under this heading we last week called attention to the important subject of female sanitary inspectors. If women would only consent to qualify themselves thoroughly for this useful branch of work, they would materially benefit themselves and their neighbours. Unfortunately, most women are lacking in foresight, and the knowledge that they will have to earn their daily bread, seldom seems incentive enough, to induce them to subject themselves to a proper course of preparation, for a future career. We feel sure that the demand for female inspectors will very soon far exceed the supply of properly trained women.

The following extract from an excellent paragraph in the *British Medical Journal* of last week deals well with this subject: "What is now urgently needed is a class of ladies thoroughly grounded in the principles of hygiene, so that with a little practical experience they may rapidly make efficient officers. The educational lectures of the Sanitary Institute given in London and so many provincial towns have been scantily attended by women, and only a select few have even presented themselves for examination. We may suggest to the hundreds of ladies, many of them of high education, directing envelopes for a small pittance, or engaged in equally unremunerative occupations, the desirability of turning their attention to this new field for employment, a field of considerable interest, and one in which much good work may be effected."

### Workhouse Infirmary Nursing Association.

THE Nineteenth-Century Art Gallery, Conduit Street, was the scene of a very pleasant evening entertainment, on Wednesday, May 31st, on the occasion of the annual gathering of the Mary Adelaide Nurses. Lady Carlisle presided, and amongst those present were Lady Wantage, the Hon. Mrs. J. G. Talbot, the Hon. Mrs. Hardcastle, Mrs. H. Bonham-Carter, Mrs. A. C. Powell, Dr. Humphreys, Mr. Bousfield, Miss Louisa Twining, Mrs. Nicholls, Dr. Savill, Miss Hughes, Miss Wood, and Miss Gill.

Miss Twining, who has done so much towards raising the standard of infirmary nursing, spoke a few words to the nurses. She said she wished photography had been in vogue forty years ago, that it might have been possible to compare the dreadful beings who answered to the name of nurses in the workhouse infirmaries of that time, with those whom she now saw before her. Then the Matron was generally the only paid woman; the rest of the attendants were paupers of the most dreadful description—creatures with bloated faces, who made a boast of how many times they had been in prison, and who actually could not be allowed to go out, their return in a state of intoxication being the sure result of freedom. Though much has been done to better these conditions, many reforms remain still to be carried out, but Miss Twining looked forward hopefully to improvements in the future, and meanwhile she trusted that the nurses would do their very best for the poor helpless creatures under their care, feeling they were thus raising the standard of nursing and helping the great army of their profession.

Medals and gratuities were presented to some of the nurses, and Dr. Saville, in a short address spoke of the very satisfactory reports the Committee received of those nurses who had been sent by the Association, to various infirmaries. He would like to take that opportunity of calling the attention of the nurses to the Royal National Pension Fund, of which they would find all particulars in the little books distributed around. It had all the advantages of a savings bank, and was as safe as the Bank of England, besides ensuring provision for old age or times of sickness.

A most enjoyable little concert followed, and it was pleasant to see how thoroughly the songs were appreciated, those given by Miss Grainger Kerr meeting with special applause. An amusing dualogue, "Fast Friends," very well done by Mrs. Gordon Shaw and Miss Ethel Robinson, caused much laughter.

Some lovely roses had been sent by Lady Wantage, and noble bunches of these were distributed amongst the nurses. A wedding present, which is being given by the Association to Princess May, was on view in the ante-room—a beautifully bound, most dainty copy of Tennyson's "Princess."

Nurses from many institutions were present—some sixty or seventy in all—St. George's-in-the-East being particularly well represented, the pink cotton dresses of the nurses belonging to that infirmary being specially bright and fresh. Looking at the pleasant intelligent faces and neat uniformed figures of the nurses scattered through the rooms last Wednesday evening, and recalling Miss Twining's description of the workhouse infirmary nurse of forty years back, it could but be felt that since so much has been done in the past there is good hope for success in the future, and we cordially echo the wish that next year and every year there may be seen a great and increasing number of nurses at this annual festival.

To everybody's regret Miss Wilson, the hon. secretary of the Association, was, through illness, unable to be present.

### Death in our Ranks.

NURSE ROBINA MILES, aged 23, died on June 1st, at Sheffield Union Infirmary, after a very short illness. She was greatly beloved by her fellow nurses and many friends.

### The Military Tournament at Islington.

### THE HOSPITAL.

OUTSIDE each entrance there waited a closely packed, orderly crowd, content to spend an hour or even two in watching for the first opening of doors which would permit a rush to be made to the unnumbered seats. The Agricultural Hall keeps all its attractions within, and only the gaily striped awnings and a flag here and there gave any hint that a fête was in progress.

Acting on the advice of an intelligent Irish policeman, we found our way to what he called a "private door," in this case represented by a pair of wide wooden gates. Through these a small party of soldiers marched out at the moment. The doors were promptly closed behind them, and we were obliged to give a humble rap with our knuckles. This signal, however, received as rapid attention as any resounding double knock at a Mayfair mansion. A courteous inquiry as to our business was made by a soldier, and on our mentioning the word "hospital" we were told that "it was close to the gate." A card sent in to the doctor on duty obtained for us immediate admittance and a kindly welcome into the smallest hospital which it has ever been our lot to inspect. Passing through the groups of officers chatting with their friends in the sunshine, we stepped straight into the tiny building which had "hospital" in plain letters on its front. Two camp bedsteads, a "field equipment," a table and two chairs, seemed the accessories to the picture of a nursing sister in the centre—she stood in her neat grey dress, scarlet cape and simple cap, attending to a new patient. The two beds were occupied, and one man's attitude suggested keen suffering, although he suffered no sound to escape his lips, his face was turned resolutely away from the light. "There's not much to see, but we're very busy," said the courteous doctor; "we've only two beds, which were meant for accidents, and now we have a good deal of sickness also to arrange for—six Australians this afternoon have come in with coughs and feverish attacks." One of these Australians occupied a chair near the table, and a splendid fellow he looked, broad shouldered and sunburnt.

We heard of several accidents that had occurred, and ventured to inquire after the fate of the hero who converted a simple into a compound fracture last week because he would not "fall out" until the conclusion of the spectacle, in the course of which a kick from another man's horse had broken the poor fellow's leg. We were told he was doing very well, and was in the Guards' hospital now.

With cordial thanks for our courteous reception we bid good-bye to the busy scene, trusting that the sick and wounded soldiers may be as well cared for in times of war as they appear to be in times of peace.

### Ceylon.

### THE CIVIL HOSPITAL, COLOMBO.

A NEW Passengers' Ward is in course of construction on a piece of ground, recently acquired by Government, adjoining the Civil Hospital, Colombo. The block will contain eight rooms and a waiting-room, surrounded on all sides by verandahs. The comfort and convenience of the patients will be thoroughly well provided for, and this addition to the hospital will materially increase its utility.

### Wants and Workers.

Will anyone give books, medical or otherwise, for a library at St. Mary's Nurses' Home, Plaistow, E.? Convalescent letters for men and women are also much wanted.

Information about Lady Roberts' Indian Nursing Fund wanted b "Ignoramus."

### Everybody's Opinion.

[*Correspondence on all subjects is invited, but we cannot in any way be responsible for the opinions expressed by our correspondents. No communications can be entertained if the name and address of the correspondent is not given, or unless one side of the paper only be written on.*]

### ROYAL NATIONAL PENSION FUND FOR NURSES.

"One of the First Thousand" writes: I am a sick pay policy holder, and desire to bring a few facts to the notice of my sister nurses. I had an illness in January and February followed by extreme debility and neuralgia. Country air, and entire rest from nursing were ordered by the doctor. Not having any home to go to, this would have been quite impossible of realization but for my being a member and sick pay policy holder of the Pension Fund, by which I was entitled to £1 per week whilst ill. I drew this sum regularly for nearly four months, until the doctor gave permission for me to resume nursing. My health is completely re-established and I am deeply grateful to those who founded this Fund: for they are true friends to nurses.

### JAPAN.

"Nurse F. S." writes: I am thinking of going out to Yokohama in the early part of next year, and shall be very much obliged to any of the numerous readers of The Hospital who can give me some information as to the nursing there. What prospect is there of a nurse getting employment in a hospital or in private families?

### QUALIFIED NURSES.

"A Certificated Nurse" writes: Under the heading "Qualified Nurses," a letter appeared in the *Lancet* of May 27th. It was signed by Mr. (or Dr.?) Cahill, who asserts "it still remains as difficult to obtain a really efficient nurse upon short notice as it has ever been within my recollection." I cannot help wondering where the gentleman goes in search of efficient nurses. I think he would have no trouble in finding one if he applied to any leading hospital or to 8, New Cavendish Street, where 200 "certificated trained nurses" are on the books of the Co-operation. Mr. Cahill complains of one whom he engaged for a patient of his, proving to have had only eight weeks' training. This nurse he took on the "recommendation by a relative of a lady who was under my care." But surely that is not the way in which most nurses are engaged. For my part, I find doctors generally very particular, and inclined to make full inquiries as to a nurse's previous experience as well as for her certificate before they would think of sending her to "a serious and somewhat complicated medical case."

### MANCHESTER INFIRMARY.

"Two Manchester Nurses" write: Will you allow two old Manchester Infirmary nurses to make a few remarks on the paragraph headed "Nurses as Scrubbers" in your issue of April 29th. We have waited in the hope that some abler advocate would take up the cudgels on behalf of the institution we are proud to own as our training school. As none have appeared, we must ourselves contradict emphatically the statement that "nurses and probationers are required to scour the floors of the wards . . . instead of attending to the patients," and that in place of receiving systematic instruction they are "left to pick up such knowledge as they can promiscuously." One of us spent five, the other four, years there, and we both feel that we can therefore speak from personal experience, not casual observation. We trust you to give our letter the publicity you gave to the extract from the *Manchester Courier*. As a matter of fact, scouring the floors is an impossibility, as, with the exception of one small ward, they are all polished. The probationers rub the floors certainly, but with no detriment to their hands, and very often to their own physical advantage. As far as practical and theoretical instruction is concerned, we think it would be hard to excel the old "M. R. I.," and we do know from experience that in some points it is unequalled. What with lectures from the honorary and resident medical staff, systematic instruction from the "Home Sister," in elementary physiology, nursing details, and bandaging; compulsory writing out notes of lectures for the inspection of the lecturer, as well as competitive examinations, we often felt as if we would have preferred the teaching to have been of a more desultory character. With regard to the practical work in the wards, we found the Sisters ever willing to teach and help any earnest nurse by showing how things should be done, and why they should be done exactly as taught. It seems hard that after five years' good work on the part of the late lady superintendent in ameliorating the condition of the nurses and elevating the tone of the hospital, the management of the institution should receive such scathing criticism. and certainly the arrangements have not deteriorated under the present *régime*. We conclude with the city motto, "*Laborare est Orare.*"

### THE ROYAL WEDDING.

Nurse Janetta writes: I like the idea of the Pension Fund Nurses having a river picnic; but as I want to have a peep at the bride, I shall be very glad if it is settled for us to have an outing after the wedding day, and not on it.

"A Sympathetic Nurse" writes: It certainly would be pleasant to commemorate the Royal wedding in some such way as was suggested in last week's Hospital. But I hope it won't be arranged for the actual day, as many nurses will wish to stay in town on purpose to witness such of the festivities as can be seen on the route of procession.

### NURSES AS PATIENTS.

"A Fever Nurse" writes: While nursing enteric fever at the Borough Hospital, Ipswich, I contracted typhoid, and I should like your readers to know how well I was cared for. The doctors were most kind and attentive, and I received all my salary whilst I was ill, and had a month's holiday afterwards to regain my strength. A personal experience of this terrible fever does indeed make a nurse sympathise with patients suffering from it. Our lives are so very busy that we may easily partially lose sight of the acute sufferings of our fellow creatures, and be tempted to think them more troublesome than they need be. I may say that all my patients have set me a good example, and I am ashamed to remember how very troublesome I was to nurse, yet no one ever grumbled at my whims or failed to gratify them.

### A PAYING COTTAGE HOSPITAL.

"E. M. N." writes: What is the best means of finding out where a small paying (or partly paying) cottage hospital is most needed, or would be most likely to succeed? Can any reader tell me of a place where there is a real want for such a thing?

### Appointments.

The Gordon Hospital.—Miss J. R. Maxwell has received the appointment of Matron at the Gordon Hospital. Miss Maxwell was trained at St. Bartholomew's Hospital and Peterborough General Infirmary, and was afterwards Matron at the Wisbech Hospital, Cambridgeshire, and at West Ham. We wish her every success in her new sphere of work.

Fulham Union Infirmary.—Miss Mary Elizabeth Shipley has been appointed Matron of the Fulham Union Infirmary. Miss Shipley was trained at St. Thomas's Hospital, and was afterwards nurse at St. Marylebone Infirmary for four years, Assistant Matron at Paddington Infirmary for four and a half years, and Matron of Stockton-on-Tees Hospital for two years and ten months. We heartily congratulate the infirmary which has had the good fortune to secure the services of this well-trained and experienced lady.

### An Association of Nurses for the Insane.

An Association of Nurses for the Insane has been talked of for some time, and it cannot be doubted that the formation of such a body might do much good. There are difficulties in its way, but they are not insuperable. The workers in our asylums form a series of isolated groups out of touch with each other and without any means of keeping up communication amongst themselves. By the public, generally, their work is misunderstood and undervalued; and many of the nurses even, still esteem their calling less highly than they might do. The spread of education among them is beginning to make its influence felt, but there is much to be done yet. Every nurse must learn to look upon the care of the insane as her life work (as the medical man looks upon his profession) and not as a mere temporary service which can be taken up and dropped again without a thought or a care. It is only after continuous application and training that she becomes completely fitted for her duties, and in proportion to her diligence and perseverance so will be her success. Many a woman joins an asylum staff with the idea that her work is little different from a species of domestic service and with little intention of continuing in it for any length of time. When every nurse must be certificated there will be no room for this type of individual, but while she exists she must be considered, and she will be a trial to those who undertake the management of such an association.

Much attention is paid to the comfort of the nursing staffs in our asylums, and in many of them pensions are given to those who, after faithful service, are incapacitated by failure of health or by accident. In addition to this the nurses should make provision for the future. Something is needed to encourage the spirit of self-help and to aid the growth of habits of thrift and foresight. If this Association supported a scheme to effect this end its appearance would be much to be desired. It would be well for its promoters to consider whether it might not be more advantageous to share in the benefits of existing schemes than to launch a new one of their own.

At the outset this Association would require a helping hand, and it is in the interest of none, so much as the medical superintendents, to give it every assistance. Its tendency would be to check that restlessness which causes so many changes among the junior members of the asylum staffs. At first, no doubt, it would be joined by many who had no intention of continuing in the work, and after being members for a year or two would weary and want to withdraw.

There are many points which might arise with regard to the training and treatment of nurses generally in the consideration of which such an Association might render valuable services to the whole body of those engaged in the care of the insane.

### Minor Appointments.

Frimley Nursing Association.—Miss Richardson has been appointed District Nurse at Frimley, in Surrey. She was trained at University College Hospital, then worked in the Incurable Hospital, Cowley, and took her L.O.S. diploma at the British Lying-In Hospital, Endell Street. We wish Nurse Richardson success in her present work.

Cheam Temporary Isolation Hospital.—Miss Sarah Jane May has been made Superintendent and Nurse of the Cheam Temporary Isolation Hospital. Miss May has nursed at the Eastern Fever Hospital, Homerton, and at Sheffield and Colchester Workhouse Infirmaries.

### For Reading to the Sick.

### TEMPTATION.

There is a story told of a monk in the 5th century who was troubled with a violent temper, which made him very unhappy. Instead of trying to curb it, however, he laid the fault on everyone he came near, imagining that if he always lived with religious people he should never be angry, so he ran away from home and entered a monastery. But even there the other monks frequently irritated him unintentionally, and he soon left them and went away into a desert, taking with him only an earthen pitcher, being sure that when he was quite alone he could not fail to find peace and rest. But, alas! he had not left himself behind. One day in returning from the spring he upset the pitcher, and the water was lost. Again and again he filled the jar, but each time some little accident caused the water to be spilt.

At last in a furious passion he dashed the pitcher against a stone, and it fell to the ground in a hundred pieces. When he came to himself he exclaimed, "O fool that I am! How can I escape the temptation that is in my nature? If there are no men to be angry with, I rage against an earthen vessel."

Like the monk we can never escape temptation, yet we can always get help by praying as our Lord has taught us, "Lead us not into temptation." The weakness of our mortal nature is so great we may be led into sin before we know it, and our care should be to look closely at all we say or do, and not be over confident, remembering St. Paul's words, "Let him that thinketh he standeth take heed lest he fall."

In sickness there are so many things to annoy and make us fretful, impatient, cross, distrustful of God and despairing, the sharpness and severity of our sufferings render our minds confused and our resolutions weak. But if in our hearts we really love God, He will with every temptation make a way of escape that we may be able to bear it. And what a comfort it is that we have the sympathy and help of our Lord Jesus Christ in every grief and trial. St. Paul once prayed most earnestly that his temptation, the thorn in the flesh, he calls it, might be taken away that he might be able to serve God more perfectly; the answer he received from the Lord was not what he wished but what was best for him. "My grace is sufficient for thee, for my strength is made perfect in weakness." The Lord's hand is not shortened, it is as powerful and far reaching in our day as it was in the great Apostle's. It was no path of flowers which the Beloved of the Father trod for thirty years in this dark world: let us face our temptations as He did His, and fight the tempter with the sword of the Spirit, which is the word of God.

### Notes and Queries.

### SPECIAL NOTICE.

The contents of the Editor's Letter-box have now reached such unwieldy proportions that it has become necessary to establish a hard and fast rule regarding Answers to Correspondents. In future, all questions requiring replies will continue to be answered in this column without any fee. If an answer is required by letter, a fee of half-a-crown must be enclosed with the note containing the enquiry. We are always pleased to help our numerous correspondents to the fullest extent, and we can trust them to sympathise in the overwhelming amount of writing which makes the new rules a necessity.

### Queries.

(146) *Information.*—Where can I find the names of secretaries and matrons of hospitals?—*Ignoramus.*

(147) *Disabled.*—How should an invalid nurse make application for the use of The Hospital Endowed Bed?—*A Policy-holder.*

(148) *Institute.*—Can a trained monthly nurse join an institute or guild?—*Monthly.*

### Answers.

(146) *Information* (*Ignoramus*).—In Burdett's "Hospital Annual," published at 428, Strand.

(147) *Disabled* (*A Policy-holder*).—Write to Editor of The Hospital, 428, Strand, and clearly state your case, and it will receive immediate attention.

(148) *Institute* (*Monthly*).—We believe you could join a guild, and some institutes would admit you, as a monthly nurse only. Of course, you could not be on the staff of an association for supplying private nurses unless you have had general training and hold a certificate.

### Sent.

### II.—NEARLY TOO LATE.

Suddenly he raised the glass to his lips, and in doing so turned his head slightly towards the door, so that his eyes fell on my figure standing within the room.

In the dim light the unexpected sight of a hospital nurse must have been a little startling. To one with shattered nerves the sight was indeed a shock.

The glass fell shivering on the tiled hearth, while the dark contents trickled slowly among the ashes.

"Who are you? Why did you come?"

"I was sent."

"Sent," he said, in a bitter tone, "to take the last hope from me." He sank back into an armchair standing near, and covered his face with his hands, trembling violently.

What could I say? I looked at the bowed form sitting before me and wondered whether it was all a dream.

Who had sent for me?

Not Colonel Philips, certainly. Not the youthful servant, whose domestic arrangements seemed so woefully upset by my unexpected arrival.

One thing was clear, I could not leave the Colonel in that state, so ill, so helpless so, alone. He looked worn out with pain; probably had not taken any food lately; was possibly suffering from want of sleep that might have affected the brain.

I placed my cloak on a chair, and lit a candle. This showed me an untasted meal pushed into a corner. I drew a large couch before the fire, and arranged the rugs as comfortably as possible. I then fetched a glass of milk from the neglected tray, and said quietly,

"I want you to drink this. It will do you good."

He looked up as if not understanding what I said.

"I have come here to take care of you," I added. "You have not had food for a long time; you must take this."

He took the glass without a word, the power to question or wonder seemed gone.

"Now I want you to lie down. I shall stay here with you to-night. You must try to sleep."

He rose with my help, evidently suffering great pain, and allowed me to cover him with rugs upon the couch.

I seated myself where he could see me, and took out my knitting to pass the time.

Gradually my patient sank into sleep, at first broken and troubled, and at length deep and refreshing.

A profound silence seemed to brood over the place broken presently by footsteps, and a low knock at the door from the little domestic, who had come to inform me that my boxes were in the spare room.

Without paying much attention to this statement, I asked her to bring me what I required in the shape of food for the night, saying I intended to stay where I was for the present.

She fetched what I wanted without any comment.

The clock had long struck the hour of four, and the faint gleams of dawn were stealing through the window, when my patient opened his eyes and spoke.

"You are still there, Sister? You are a Sister, are you not? I remember one like you when I was in the Crimea, long before you can remember. That was when my leg was first bad, and she nursed me."

"I have had bad dreams, Sister," he said at last. "Bad dreams. They never left me night or day. God knows what torments I have had. I don't know why you came, or who sent you; but you have kept me from the darkest dream of all."

He turned his eyes to the window with the growing light. The new dawn seemed a type of the newer life and hope within him. I felt it would do him good to talk. I begged him to tell me why he had been so much alone. "Had he no relations who could come —— "

He interrupted me. He had a son—the best son a man ever had. He lived near—only the other end of the village, but he had been taken ill some weeks ago, and had been lying between life and death. "And I could not get to him," he said. "My old wound broke out again. It was agony. I could hardly move; for days I hardly tasted food, and at night I never slept. An old soldier does not like to complain; and they were all so anxious about Roy that they had not time to come here. I thought I could battle through with it. I have never been beaten yet; but it got the better of me. There was one dream," he shuddered at the recollection, "which kept coming, and seemed to drive me mad, and then last night--" He paused and put his hand to his head. "It is so confused. You came in Sister?"

"Yes."

"And I was standing near the fire? Had I—had I a glass in my hand?"

"Yes."

A pause—"I did not drink out of it; I saw you and it fell, was that it?"

"Yes."

A long silence.

He put one hand on my arm, and covered his face with the other, and remained so; whether sleeping or thinking I could not tell.

Early in the morning the maid came to inform me that a lady wished to speak to me, and I at once went to the entrance.

"I have no time to come in," she said, "but I thought it better to explain the mistake made last night. I telegraphed for a nurse for my husband, Mr. Roy Philips, the Colonel's son, and the cabman brought you here instead. Fortunately, my husband was better last evening, or the delay might have been serious. I hope you found a place to sleep in. You have not seen the Colonel? He very seldom sees strangers now."

I hesitated as to how much I should tell her. Her kindly, sensible face prompted me to tell her everything, and in as few words as possible I let her know what I had found.

She reproached herself bitterly for having left the old man so much. "He never said a word about himself. He is the most unselfish man that ever lived, and we were so wrapped up in Roy. Oh, how can I thank you for all that you have done? I meant to take you back with me, but we can manage very well. I will wire to town for another nurse; you must not leave father, please. If we had only known! No one guessed what he was suffering."

And I did not leave my patient whom I had found "by mistake" for many weeks. His recovery was slow, but gradually the old health and vigour came again.

When Roy was able to come and see us his gratitude knew no bounds.

"You saved my father's life, Sister," he repeated over and over again.

"My son, she has done more than that," said the old Colonel. "She has saved an old soldier's honour." He added, with a voice not quite under command and a solemn smile on his dear old face—

"Ah! Sister, an old soldier ought to have known better than to try to leave his post before his Commanding Officer sends for him."

Dagmar.

### Presentation.

Nurse Annie Gillie, having recently completed her eighteenth year of residence at the Royal Infirmary, Newcastle-on-Tyne, her fellow-workers showed their kindly appreciation of her long service by presenting her with a travelling bag, a clock, and other useful gifts.

### The Book World for Women and Nurses.

[We invite Correspondence, Criticism, Enquiries, and Notes on Books likely to interest Women and Nurses. Address, Editor, THE HOSPITAL (Nurses' Book World), 428, Strand, W.C.]

HOSPITAL SISTERS AND THEIR DUTIES. By EVA C. E. LÜCKES, Matron of the London Hospital. (Published 428, Strand. Price 2s. 6d.)

The third editon of Miss Lückes' admirable book, "Hospital Sisters and their Duties," will be welcomed by many readers. An additional chapter gives special value to the present issue, the former heading of Chapter IV., "The Training of Probationers," being now changed to "Practical Training of New Probationers," whilst the recently inserted pages treat particularly of the "Training of more Advanced Probationers." Certainly the book will be read with immense interest by many persons who may never have done a day's work in hospital, and yet honestly desire to learn by what process an enthusiastic amateur is developed into an accomplished head nurse or sister. Elaborate attention to details has evolved a perfect system which comes as a marvellous revelation to general readers. Everything in any way connected with trained nursing has to be thoroughly and intelligently taught nowadays. Nothing is left to chance; which sounds strange to the ears of people who have hitherto believed in ward duties "coming naturally" to probationers. The original edition of "Hospital Sisters" is well known to many of our readers, yet there may be some who hardly comprehend that the book is as suitable for nurse pupils as for the sisters themselves. Miss Lückes' clever theoretical instruction shows fresh light on the relationship which should exist between all classes of hospital workers, and many a novice might be spared uncomfortable experiences if she understood from the first, the actual position of the sister or head nurse. There is "a natural inclination in young nurses, says Miss Lückes, to "enter the wards with an impression that the sister under whom they are to serve must be a woman who cannot fail to win their deepest respect, if not their cordial admiration and affection; such a woman as they may humbly hope to become in a distant future, when they have been taught the innumerable things that they need to learn, and have worked, as they mean to work, with the view of one day deserving the title of a thoroughly trained nurse." But there are also probationers who seem inspired to criticise everything which they do not understand, and their hasty judgments would be considerably modified if they perused this little book. The duties of assistant sisters is also very thoroughly gone into in the present edition. The other chapters remain in their original form, and certainly no improvement could be suggested in these; from the "Domestic Management of Wards" to the delightful pages treating of the "Relationship of Sisters to their Patients," there is hardly a line which will not help to smooth the way of some newly-appointed matron, trained nurse, or earnest probationer, and the new volume cannot fail to maintain the popularity already accorded to former editions.

### THE BOOK WORLD FOR WOMEN AND NURSES—*continued.*

### MAGAZINES FOR JUNE.

An amusing article in Temple Bar on Lady Mary Wortley Montague recalls the fact that the anti-vaccination agitation has not by any means been the outcome of our own day only. It was at Adrianople that she directed her attention to inoculation for small-pox, a set of old women there performing the operation every autumn. "People send to one another to know if any of their family have a mind to have the small-pox. They make parties of fifteen or sixteen for the purpose. . . The French Ambassador says that they take the small-pox here as they take the waters in other countries. There is no example of anyone that has died in it." She was brave enough to have her little son inoculated, and later on her daughter. But "she vowed she would never have attempted it if she had foreseen the vexation, the persecution, the obloquy it brought upon her. The clamours raised against the practice and, of course, against her were beyond belief. The faculty rose in arms to a man, foretelling the most disastrous consequences. The clergy descanted from their pulpits on the impiety of thus seeking to take events out of the hands of Providence. The common people hooted at her as an unnatural mother who had risked the lives of her children. . . The four great physicians deputed by Government to watch the progress of inoculation in the case of Lady Mary's daughter, betrayed such incredulity as to its success, and such an unwillingness to have it succeed, that she never liked to leave the child alone with them one second, lest it should suffer from their interference."

Mr. Wolff gives rather a cynical account in the Gentleman's Magazine of the gospel of health known as the Kneipp cure, and carried on at Wörishofen under the direction of Father Kneipp himself. It is far from being an ordinary water cure, though water plays undeniably the first part in the system. Early morning walks barefoot in wet grass, or snow, if procurable, are enjoined on the Kneippist; if the grass is not wet enough with dew or rain for his requirements, the hose must be employed to drench it thoroughly and produce the right degree of chill in the patient preparatory to the "première réaction" on which much of the efficacy of the cure depends. The day is spent in getting alternately cooled down by paddling barefoot in rivers or ditches, by the administration of "knee gushes," cold "shawls," or "Spanish mantles" (varieties of "packing"), and having the circulation restored by hot blankets and violent exercise. In addition to these delights Father Kneipp nourishes his adherents on a variety of curious concoctions of his own brewing. Rye bread toast and water, bran broth and honey, roast barley, and an unlimited supply of strawberries seem the principal items in the bill of fare. But in between meals the patient must drink his way steadily through a course of herb teas warranted to cure anything you please. Violet tea sounds an innocent cure for consumption and eye-bright tea a poetic one for sore eyes. But what shall we say of knot grass and mistletoe, of hay-loft sweepings, juniper berries, rosin, wormwood, and chalk? The prospect of such refections will go far to make the intending Kneippist pause and reflect whether the dew on his lawn may not suffice him without a visit to Wörishofen.

